# Medicines information provided by pharmaceutical representatives: a comparative study in Australia and Malaysia

**DOI:** 10.1186/1471-2458-10-743

**Published:** 2010-11-30

**Authors:** Noordin Othman, Agnes I Vitry, Elizabeth E Roughead, Shaiful B Ismail, Khairani Omar

**Affiliations:** 1 Quality Use of Medicines and Pharmacy Research Centre, School of Pharmacy and Medical Sciences, University of South Australia, Adelaide, Australia; 2 Kulliyyah of Pharmacy, International Islamic University Malaysia, Kuantan, Pahang, Malaysia; 3 Department of Family Medicine, School of Medical Sciences, Universiti Sains Malaysia, Kelantan, Malaysia; 4 Department of Family Medicine, Faculty of Medicine, Universiti Kebangsaan Malaysia, Kuala Lumpur, Malaysia

## Abstract

**Background:**

Pharmaceutical representatives provide medicines information on their promoted products to doctors. However, studies have shown that the quality of this information is often low. No study has assessed the medicines information provided by pharmaceutical representatives to doctors in Malaysia and no recent evidence in Australia is present. We aimed to compare the provision of medicines information by pharmaceutical representatives to doctors in Australia and Malaysia.

**Methods:**

Following a pharmaceutical representative's visit, general practitioners in Australia and Malaysia who had agreed to participate, were asked to fill out a questionnaire on the main product and claims discussed during the encounter. The questionnaire focused on provision of product information including indications, adverse effects, precautions, contraindications and the provision of information on the Pharmaceutical Benefit Scheme (PBS) listings and restrictions (in Australia only). Descriptive statistics were produced. Chi-square analysis and clustered linear regression were used to assess differences in Australia and Malaysia.

**Results:**

Significantly more approved product information sheets were provided in Malaysia (78%) than in Australia (53%) (P < 0.001). In both countries, general practitioners reported that indications (Australia, 90%, Malaysia, 93%) and dosages (Australia, 76%, Malaysia, 82%) were frequently provided by pharmaceutical representatives. Contraindications, precautions, drug interactions and adverse effects were often omitted in the presentations (range 25% - 41%). General practitioners in Australia and Malaysia indicated that in more than 90% of presentations, pharmaceutical representatives partly or fully answered their questions on contraindications, precautions, drug interactions and adverse effects. More general practitioners in Malaysia (85%) than in Australia (60%) reported that pharmaceutical representatives should have mentioned contraindications, precautions for use, drug interaction or adverse effects spontaneously (P < 0.001). In 48% of the Australian presentations, general practitioners reported the pharmaceutical representatives failed to mention information on PBS listings to general practitioners.

**Conclusions:**

Information on indications and dosages were usually provided by pharmaceutical representatives in Australia and Malaysia. However, risk and harmful effects of medicines were often missing in their presentations. Effective control of medicines information provided by pharmaceutical representatives is needed.

## Background

Global pharmaceutical sales in 2008 were estimated at US$ 773 billion [1]. Sales growth has nearly doubled since 2001 [1]. Pharmaceutical companies secure their market share by using promotional techniques, including pharmaceutical detailing. Concerns have been raised about the quality of medicines information provided by pharmaceutical representatives to doctors, with studies consistently showing that benefits of medicines are promoted and risk information is less commonly provided [2-4].

In 1997, a systematic review [5] found four studies that examined medicines information provided by pharmaceutical representatives to doctors in three developed countries; Finland, the US and Australia. In Finland, two studies of 46 and 69 presentations by pharmaceutical representatives in hospitals and outpatient clinics were published in 1977 [2] and 1988 [6] respectively. Medical students and doctors acted as participant observers and filled out questionnaires after the interactions [2,6]. In both studies, indications (range per study 90-94%) and generic names (range per study 62-78%) were usually mentioned by pharmaceutical representatives. However, side effects (range per study 27-41%) and contraindications were less frequently provided (range per study 25-34%).

In 1995, statements made by pharmaceutical representatives during 13 presentations to medical students and doctors in teaching hospitals in the US were audiotaped and analysed for accuracy [3]. Eleven percent of 106 statements were rated inaccurate and provided a favourable impression towards the promoted drug [3]. An Australian study audiotaped and analysed 16 detailing presentations from December 1992 to February 1994 [4]. Adverse effects were mentioned in 27% of the presentations, contraindications were never mentioned, and use in pregnancy was only mentioned on one occasion. Information on adverse reactions appeared to be associated with claims aimed at minimizing the apparent risk associated with product use rather than detailing the possible adverse effects [4].

A survey in France from 1991 to 2005 used a network of doctors to monitor pharmaceutical representatives' presentations [7]. Doctors were recruited from among La revue Prescrire's subscribers and completed questionnaires after sales detailing by pharmaceutical representatives. Consistent results were noted in the 15 year survey. Indications were commonly provided (range per year, 64-81%) by pharmaceutical representatives. However, contraindications, precautions, drug interactions and side effects were less frequently mentioned (range per year, 9-35%) [7].

Despite differences in methodology, the results of all these studies [2-4,6,7] suggest that pharmaceutical representatives did not provide balanced medicines information to doctors. Indications and generic names were commonly mentioned, but risk information was often omitted.

In Australia, the interactions between pharmaceutical representatives and doctors are governed by the pharmaceutical companies association, Medicines Australia Code of Conduct [8], which complements the standards set by the Australian government Therapeutic Goods Act 1989 [9]. Likewise, in Malaysia, pharmaceutical representatives' activities are controlled by the Pharmaceutical Association of Malaysia (PhAMA) Code of Conduct [10] and comply with the requirements of the Medicine (Advertisement and Sale) Act 1956. Both codes require that provision of medicines information made by pharmaceutical representatives be balanced, accurate, correct, fully supported by the product information, literature or data on file or appropriate industry sources, where these do not conflict with the product information [8,10].

Pharmaceutical representatives in Australia and Malaysia are required to have sufficient medical and technical knowledge to present information on the company's products in a current, accurate and balanced manner [8,10]. Medicines Australia requires that all pharmaceutical representatives complete an endorsed Medicines Australia education program for medical representatives [8]. In Malaysia, pharmaceutical representatives need to obtain a minimum educational level of a Sijil Tinggi Pelajaran Malaysia (equivalent to year 12)[11]. Additionally, PhAMA offers non-compulsory training for pharmaceutical representatives [12]. In both countries, the regulation of pharmaceutical representatives' activities is based on a complaints mechanism [8,10]. Neither Medicines Australia nor PhAMA proactively monitor pharmaceutical representatives' activities [8,10].

In 1986, The 39th World Health Assembly called on governments to implement a National Medicinal Drug Policy [13]. Many countries, including Australia [14] and Malaysia, have developed national medicines policies [13,14]. The national medicines policies provide a pharmaceutical system framework to ensure equitable and timely access to high quality medicines to support rational use of medicines. In both countries, health care is delivered by private and government sectors. In Australia, the government heavily subsidizes medicines in both sectors through the Pharmaceutical Benefits Scheme (PBS) and the Repatriation Pharmaceutical Benefits Scheme (RPBS) [15]. The consumers pay a proportion of total costs out of pocket. In Malaysia, medicines in the public health care services are subsidized by the government with minimum fees being charged to the public, whereas in the private sector patients are required to pay full price for their medications [16,17]. However, compared to Australia, several aspects of the Malaysian national medicine policy have not been implemented yet. In particular, there has been no public examination, either by non-governmental or governmental organisations, of the success of the current regulatory system in controlling pharmaceutical promotion. In Australia, several studies have assessed the quality of pharmaceutical promotion over time [4,18-20]. Watchdog organisations such as Healthy Skepticism and CHOICE are repeatedly raising concerns about misleading drug promotion [21,22] and the adequacy of Medicines Australia's code of conduct is reviewed regularly by the Australian Competition and Consumer Commission to ensure compliance with the Australian Trade Practices Act [23].

The Malaysian pharmaceutical market is growing. From 1985 to 2007 a total of 175,746 products were approved by the Malaysian Drug Control Authority [24]. This has made the market more competitive [25] and pharmaceutical companies appear to be heavily promoting their medicines to Malaysian health professionals [26]. Given the absence of a comprehensive independent source of prescribing information in Malaysia, Malaysian doctors may be more likely to rely on medicines information provided by pharmaceutical companies.

To our knowledge, no study has assessed the quality of medicines information provided by pharmaceutical representatives to doctors in Malaysia and there is no recent evidence from Australia. We aimed to compare the quality of medicines information provided by pharmaceutical representatives to doctors in Australia and Malaysia, in particular the provision of information on potential harmful effects of a medicine including contraindications, precautions, drug interactions and adverse effects.

## Methods

This study was a prospective observational study of doctor-pharmaceutical representatives' encounters. It was conducted from 15/8/07 to 15/4/2009 (20 months). Doctors in primary care settings in Australia and Malaysia, who met pharmaceutical representatives in their regular practice and were practicing at least 25 hours per week during the study period, were invited to participate. This study was approved by the Human Research Ethics Committee of the University of South Australia, the Universiti Sains Malaysia (USM) and the Universiti Kebangsaan Malaysia (UKM).

Doctors were asked to monitor four to ten encounters with pharmaceutical representatives. A doctor-pharmaceutical representative encounter was defined as one meeting between doctor(s) and pharmaceutical representative(s) which happened at the doctor's surgery. 

Both one-on-one and group presentations were assessed. The meeting was based on an appointment and had to be longer than one minute. "Corridor" meetings with pharmaceutical representatives were not considered suitable for this study. Doctors were encouraged to see as many pharmaceutical representatives during the study period as usual.

### Sample size calculation

La Revue Prescrire, which organised a pharmaceutical representatives' monitoring network in France for 15 years, reported that on average, adverse effects were mentioned in 32% of sales representatives' promotional presentations [27]. In this study, an absolute difference between pharmaceutical sales detailing in Malaysia and Australia of 20% in the availability of information on particular adverse effects was considered a substantive difference. A sample size of 66 encounters per study arm would detect this degree of difference with 80% power at α = 0.05.

In Canada, it has been reported that doctors received a mean of 5.6 visits per month from sales representatives (range 0-28) and a mean of 2.2 products per visit [28]. This study assumed a similar situation in Australia and Malaysia. If doctors recorded information on four presentations, a sample size of 20 doctors per study arm would provide 80 observations. Data collection could be completed within four to five weeks per physician.

### Recruitment of general practitioners

#### Australia

Two mechanisms were employed to recruit doctors in Australia.

Eleven divisions of general practices in different states of Australia contacted general practitioners on our behalf. These organizations are members of the network of Australian Divisions of General Practice and funded by the Australian Government Department of Health and Ageing [29].

Doctors who were Healthy Skepticism [21] subscribers (HSS) were invited to participate in the study and to nominate two doctors who could also be willing to participate. The nominated doctors were also invited to participate. Healthy Skepticism is an international non-profit organisation aiming to improve health by reducing harm from misleading drug promotion [21]. This organisation uses research, education and advocacy to improve drug promotion by the pharmaceutical industry. Healthy Skepticism subscribers have an interest in research on pharmaceutical promotion and were considered more likely to participate in the study.

#### Malaysia

In Malaysia, primary care treatment to the public is provided by three different types of doctors. These are general practitioners in private clinics, family medicine doctors who are undergoing specialist training in teaching hospitals, and family medicine specialists in teaching hospitals or private clinics. All types of primary care providers were included in this study.

Doctors from family medicine departments in two teaching hospitals in Malaysia, Universiti Sains Malaysia, Kota Bharu (USM), Universiti Kebangsaan Malaysia, Kuala Lumpur (UKM), general practitioners in private practices in Kota Bharu and Kuala Lumpur were invited to participate in this study.

Doctors who agreed to participate were asked to nominate two general practitioners from private practice who could also be willing to participate in this study. The nominated doctors were also invited to participate in the study.

The Malaysian Medical Association (MMA) [30] invited its members in rural and urban areas to participate in the study. MMA is a professional body representing Malaysian doctors, which focuses its activities on enhancing healthcare in Malaysia.

All doctors from the Australian and Malaysian lists were invited to participate in this study by a letter or an email. We invited 3038 doctors in Australia and 819 doctors in Malaysia.

Of 3038 Australian doctors that were invited, 2955 were contacted by general practitioner divisions, 70 by Healthy Skepticism and 13 via nominations from general practitioners. In Malaysia, 119 general practitioners were invited from family medicine departments, while 694 were contacted via MMA and six were invited based on recommendations from general practitioners. A reply letter with a prepaid envelope was attached to the invitation letter. A follow up letter or email was sent if no reply was received within two weeks. In Australia, we posted a brief advertisement in the newsletter provided by the South Australia Divisions of General Practice Inc. to 1738 general practitioners in the divisions.

An information sheet was sent to doctors who had agreed to participate in the study. Participating doctors were asked to provide personal details on gender, age, years in practice, postgraduate qualifications, number of doctors in practice and average number of pharmaceutical representatives met every week. Each doctor was assigned an identifying code. Pharmaceutical representatives were made aware of the study and were asked for consent to participate. Only doctors were allowed to get written consent from pharmaceutical representatives.

Following a pharmaceutical representative's visit, doctors filled out a questionnaire focusing on the main product and claims discussed during the encounter. The main product refers to the product that was approved by the authorities to be marketed and was given most attention by the pharmaceutical representative in a single encounter. Doctors were required to report their own assessment of the prescribing frequency of the products promoted. The questionnaire focused on provision of product information, including indications, adverse effects, precautions, contraindications and the provision of information on the listing of a medicine on the Pharmaceutical Benefit Scheme (PBS), the Australian public insurance scheme [31]. "New medicine" was defined as a medicine that general practitioners believed would be marketed soon or had been marketed recently. "Old medicine" referred to a medicine that general practitioners believed had been marketed for a period of time.

Doctors were asked to keep the completed questionnaires in a secure place and send them to the researchers every two weeks in a prepaid reply envelope. The questionnaire was based for its major part on questionnaires developed in previous studies [4,7,32]. The face validity of the questionnaire was assessed by six experts in the field of study and doctors in Australia, Malaysia and Canada. The questionnaire was then modified based on their comments.

Data entry was undertaken using SPSS database version 17.0. Chi-square analysis was used to assess differences in the provision of product information given by pharmaceutical representatives to doctors in Australia and Malaysia. As there were multiple observations for each general practitioner, additional clustered linear regression was conducted with STATA 10 to assess if doctors reported information provided to them differently. The Bonferroni correction for multiple comparisons was applied.

## Results

**Figure 1:**
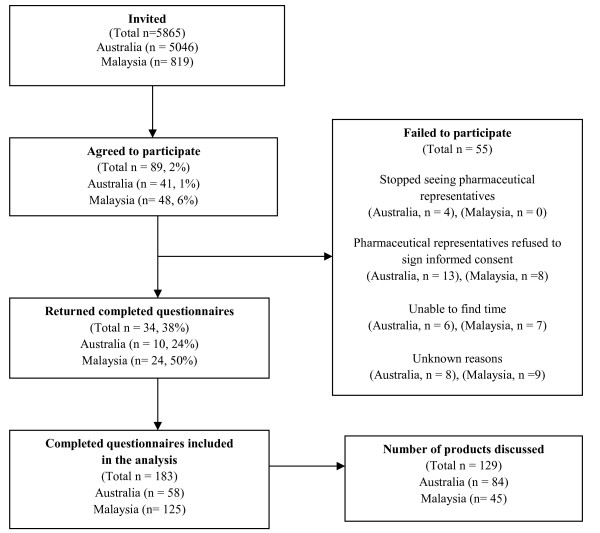
**Recruitment and participation of general practitioners**.

A total of 89 general practitioners agreed to participate into the study and 34 returned 183 completed questionnaires (Figure 1). More general practitioners agreed to participate and returned completed questionnaires in Malaysia than in Australia. In Malaysia, from 127 returned questionnaires, two were excluded, as the main products detailed were a baby milk formula and a fermented milk drink. In Australia, of 10 general practitioners who returned the completed questionnaires, three of them were Healthy Skepticism members. On average, each general practitioner completed six and five questionnaires in Australia and Malaysia respectively.

Australian and Malaysian general practitioners were aged 45-54 years old (70%) and 35-44 years old (50%) respectively (Table 1). The majority of Australian (70%) and Malaysian (75%) general practitioners had postgraduate qualifications and met one to three pharmaceutical representatives every week. The majority of general practitioners in Australia (60%) and all general practitioners in Malaysia practiced in urban locations.

**Table 1:** Characteristics of general practitioners

Characteristics		Australia n/10 (%)	Malaysia n/24 (%)
Gender	Male	5 (50)	14 (58)

	Female	5 (50)	10 (42)

		^P = 0.85	

Age	< 35 years	0	3 (13)

	35-44 years	1 (10)	12 (50)

	45-54 years	7 (70)	7(29)

	> 54 years	2 (20)	2 (8)

		^P = 0.01	

Years in practice	Less than 5 years	1 (10)	3 (12)

	5-10 years	0	5 (21)

	10-20 years	4 (40)	12 (50)

	Over 20 years	5 (50)	4 (17)

		^P = 0.02	

Postgraduate qualifications	Fellowship and postgraduate	6 (60)	8 (33)

	Fellowship only	2 (20)	0

	Postgraduate only	1 (10)	10 (42)

	None	1 (10)	6 (25)

		^P = 0.02	

Site of practice	Urban	6 (60)	24 (100)

	Rural	4 (40)	0

		^P = 0.001	

Number of doctors in practice	1	0	8 (33)

	2-5 doctors	6 (60)	4 (17)

	More than 5 doctors	4 (40)	12 (50)

		^P = 0.03	

Average number of pharmaceutical representatives met every week	1-3	7 (70)	14 (58)

	4-6	3 (30)	10 (42)

		^P = 0.09	

^ P-value (Comparison Australia and Malaysia)

A total of 45 and 84 main medicines were discussed in pharmaceutical representatives' presentations in Australia and Malaysia respectively. The most frequently promoted medicines in both countries were for cardiovascular diseases. More new medicines were discussed in Malaysia than in Australia (P < 0.001) (Table 2). The majority of presentations in both countries were personal presentations (Australia, 98%, Malaysia, 98%).

**Table 2:** Presentations details

Variables	Details	Australia n/58 (%)	Malaysia n/125 (%)
Type of main product discussed	New medicine	9 (16)	41 (33)

	Old medicine with new information/indication	18 (31)	54 (43)

	Old medicine with general information	31 (53)	30 (24)

		^ P < 0.001	

Type of presentation	Personal	50 (86)	122 (98)

	Group	8 (14)	3 (2)

		^ P = 0.01	

Current usage of the main product	Low*	14 (24)	33(26)

	Medium^&^	26 (44)	43 (34)

	High#	9 (16)	8 (7)

	Non user**	9 (16)	41 (33)

		^ P = 0.03	

Length of discussion	1-5 minutes	16 (27)	42 (33)

	> 5-10 minutes	30 (52)	55 (44)

	> 10-20 minutes	10 (18)	26 (21)

	> 20 minutes	2 (3)	2 (2)

		^ P = 0.65	

* Low = rarely used, ^&^Medium = seldomly used, # High = commonly used, **Non user = never used, ^ P-value (Comparison Australia and Malaysia)

When old medicines with new information or indications were presented, the majority of general practitioners in Australia (61%) and Malaysia (52%) reported being medium users of the products. In all presentations where a medicine was classified as "new", general practitioners in both countries reported that they were non users. About half of discussions lasted from five to ten minutes (Australia, 52%, Malaysia, 44%).

Approved product information sheets were provided in more than half of the presentations (Australia, 53%, Malaysia, 78%), and more commonly in Malaysia than in Australia (P < 0.001) (Table 3).

**Table 3:** Provision of information

Information		Australia n/58 (%)	Malaysia n/125 (%)
Approved product information sheet given	Yes	31 (53)	97 (78)

	No	27 (47)	28 (22)

		^P < 0.001	

Indications	Yes	52 (90)	116 (93)

	No	6 (10)	7 (5)

	Unsure	0	2 (2)

		^*P = 0.33	

Dosage	Yes	44 (76)	103 (82)

	No	14 (24)	22 (18)

		^P = 0.30	

Contraindications	Yes	19 (33)	50 (40)

	No	37 (64)	72 (58)

	Unsure	2 (3)	3 (2)

		^*P = 0.37	

Precautions	Yes	21(36)	38 (30)

	No	34 (59)	82 (66)

	Unsure	3 (5)	5 (4)

		^*P = 0.40	

Drug interactions	Yes	19 (33)	31 (25)

	No	38 (65)	89 (71)

	Unsure	1 (2)	5 (4)

		^*P = 0.30	

Adverse effects	Yes	24 (41)	45 (36)

	No	33 (57)	73 (58)

	Unsure	1 (2)	7 (6)

		*P = 0.61	

Did the representative answer your questions on contraindications, precautions, drug interactions, adverse effects?	Yes	34/45 (76)	60/105 (57)

	No	2/45 (4)	7/105 (7)

	Partly	9/45 (20)	38/105 (36)

	No question asked	13	20

		^^&^P = 0.10	

Given the nature of the drug detailed, do you think the representative should have mentioned contraindications, precautions for use, drug interaction or adverse effects spontaneously?	Yes	35 (60)	106 (85)

	No	23 (40)	19 (15)

		^P < 0.001	

*Chi square test for questions that included variable "unsure" was calculated based on categorical variables "Yes" and "No" only.

^&^Chi square test for a question with a variable "no question asked" was calculated based on categorical variables "Yes", "No" and "Partly" only.

^ P-value (Comparison Australia and Malaysia)

In both countries, general practitioners reported that information on indications (Australia 90%, Malaysia 93%) and dosages (Australia 76%, Malaysia, 82%) was frequently given by pharmaceutical representatives for all types of products discussed (Table 3 and 4). General practitioners indicated that information on negative aspects such as contraindications, precautions, drug interactions and adverse effects (Australia 41%, Malaysia 36%) was less commonly provided. However, this information was provided more frequently for new medicines than for old medicines.

**Table 4:** Provision of information for type of main product discussed

Information		New medicine		Old medicine with new information/indication		Old medicine with general information	
Type of product		Australia n/9 (%)	Malaysia n/41 (%)	Australia n/18 (%)	Malaysia n/54 (%)	Australia n/31 (%)	Malaysia n/30 (%)

Indications	Yes	8 (89)	39 (96)	17 (94)	49 (91)	27 (87)	28 (93)

	No	1(11)	1(2)	1 (6)	4 (7)	4 (13)	2 (7)

	Unsure	0	1(2)	0	1 (2)	0	0

				^*P = 0.72			

				^&^*P = 0.56			

Dosage	Yes	7 (78)	35 (85)	14 (78)	45 (83)	23 (74)	23 (77)

	No	2 (22)	6 (15)	4 (22)	9 (17)	8 (26)	7 (23)

				^P = 0.95			

				^&^P = 0.62			

Contraindications	Yes	5 (55)	24 (59)	4 (22)	20 (37)	10 (32)	6 (20)

	No	3 (33)	17 (41)	13 (72)	32 (59)	21 (68)	23 (77)

	Unsure	1 (12)	0	1 (6)	2 (4)	0	1 (3)

				^*P = 0.15			

				^&^*P = 0.01			

Precautions	Yes	4 (45)	17 (41)	5 (28)	14 (26)	12 (39)	7 (23)

	No	4 (45)	23 (57)	12 (66)	38 (70)	18 (58)	21 (70)

	Unsure	1 (12)	1 (2)	1 (6)	2 (4)	1 (3)	2 (7)

				^*P = 0.59			

				^&^*P = 0.19			

Drug interactions	Yes	5 (55)	16 (39)	3 (16)	11 (20)	11 (35)	4 (13)

	No	4 (45)	23 (56)	14 (78)	41 (76)	20 (65)	25 (84)

	Unsure	0	2 (5)	1 (6)	2 (4)	0	1 (3)

				^*P = 0.14			

				^&^*P = 0.02			

Adverse effects	Yes	6 (67)	23 (57)	7 (39)	16 (30)	11 (35)	6 (20)

	No	3 (33)	17 (41)	11 (61)	34 (63)	19 (62)	22 (73)

	Unsure	0	1 (2)	0	4 (7)	1 (3)	2 (7)

				^*P = 0.26			

				^&^*P = 0.01			

*Chi square test for questions that included variable "unsure" was calculated based on categorical variables "Yes" and "No" only.

^ P-value (Comparison between type of products: Australia)

^&^ P-value (Comparison between type of products: Malaysia)

The clustered regression analysis found similar results to the chi square analysis: there were no significant differences between countries regarding the availability of medicines information on indications (p = 0.69), dosages (p = 0.32), contraindications (p = 0.38), precautions (p = 0.53), drug interactions (p = 0.24) and adverse effects (p = 0.35).

The comparison of the provision of medicines information reported by Healthy Skepticism subscribers (HSS) and non-subscribers (HNS) suggested different trends. Reports on provision of medicines information on indications (HSS 89%, HNS 90%, p = 0.94), dosages (HSS 56%, HNS 79%, p = 0.12) and contraindications (HSS 22%, HNS 36%, p = 0.42) were not significantly different. However, Healthy Skepticism subscribers reported that information on precautions (HSS 0%, HNS 46%, p = 0.01), interactions (HSS 0%, HNS 40%, p = 0.02), and adverse effects (HSS 11%, HNS 48%, p = 0.04) was less likely to be provided by pharmaceutical representatives than non Healthy Skepticism providers.

General practitioners in Australia and Malaysia indicated that in more than 90% of presentations, pharmaceutical representatives partly or fully answered their questions on contraindications, precautions, drug interactions and adverse effects. More general practitioners in Malaysia (85%) than in Australia (60%) reported that pharmaceutical representatives should have mentioned contraindications, precautions for use, drug interaction or adverse effects spontaneously (P < 0.001).

Overall, about half of the presentations did not include any information on potential harm of the medicine, such as adverse effects, interactions or contraindications (Australia 50%, Malaysia, 46%). Of the presentations where negative effects were missing, pharmaceutical representatives provided approved product information sheets in about half of the presentations (Australia, 41% Malaysia, 68%). Information on harm was missing in more than one-third of presentations in which general practitioners believed pharmaceutical representatives should have mentioned it (Australia, 34%, Malaysia 41%). Approximately one-fifth of general practitioners in both countries (Australia 22%, Malaysia 16%, p = 0.13) did not ask pharmaceutical representatives any questions on contraindications, precautions, drug interactions or adverse effects.

In 48% of the Australian presentations, general practitioners reported the pharmaceutical representatives failed to mention information on PBS listings to them.

## Discussion

Similar trends in the provision of medicines information by pharmaceutical representatives were reported by general practitioners in Australia and Malaysia. Information on indications and dosages was usually provided in pharmaceutical representatives' presentations. However, contraindications, precautions, drug interactions and adverse effects were often missing in both countries. The imbalance of medicines information provided by pharmaceutical representatives is consistent with the findings of previous studies [2-4,6,7] and raises the question of the effectiveness of the existing regulatory systems to oversee medicines information provided by pharmaceutical representatives. In both countries, unlike for printed advertisements, the requirements set by the pharmaceutical companies' codes of conduct on the provision of medicines information by pharmaceutical representatives are vague [8,10]. Medicines Australia and PhAMA codes of conduct require that printed advertisement on medicines must disclose the properties of medicines based on the approved product information. For instance, printed advertisements shall include information on dosages, indications, side effects, contraindications and precautions [8,10]. No similar provision is available for verbal statements made by pharmaceutical representatives [8,10].

The lack of difference in quality of information provided between Australia and Malaysia may seem surprising, as it could have been expected that the enforcement of the regulation of pharmaceutical promotion would be stronger in Australia than in Malaysia. However, unlike other promotional activities, presentations done by pharmaceutical representatives are not monitored by Medicines Australia.

Australian general practitioners reported that approved product information sheets were not provided in about half of the presentations, despite it being a requirement of Medicines Australia that wherever a promotional claim is made, pharmaceutical representatives must offer the current approved product information sheets, or make reference to them in any printed promotional provided [8]. Although it is not required by the PhAMA code of conduct [10], the approved product information was provided voluntarily by Malaysian pharmaceutical representatives more often than in Australia.

The proportion of general practitioners that think pharmaceutical representatives should have mentioned information on harm spontaneously was higher in Malaysia (85%) than in Australia (60%), and also higher than reported in France (77%) [7]. This may reflect higher needs of Malaysian practitioners, who may have less ready access to medicines information than Australian or French practitioners.

The incompleteness of medicines information provided by pharmaceutical representatives in Malaysia is of concern. As no comprehensive independent source of medicines information is available in Malaysia, the government needs to proactively develop suitable and effective services to provide independent medicines information to doctors. The provision of an independent information service by the National Prescribing Service (NPS) [33] in Australia could be a model for Malaysia. NPS is an independent, non-profit organisation funded by the Australian government which provides doctors with educational materials on medicines and organised academic detailing visits [33]. Although the program is small in comparison to the large amount of money spent in pharmaceutical promotion [34,35], evidence has shown that such activities provide savings on government spending on medicines [36], improve prescribing patterns [37] and reduce prescription errors [38]. The omission of essential prescribing information in pharmaceutical representatives' presentations in Australia, highlights the need for doctors to rely more on independent sources of information.

We noted that information on the negative effects of medicines was provided more frequently for new medicines than for old medicines. However, the small sample size limits the statistical power of the comparison between types of medicines. The results have to be confirmed with a larger study.

In this study, we actively asked representatives for consent. This may have led to the pharmaceutical representatives changing their behaviour. However, we consider any bias this may have introduced would have led to pharmaceutical representatives improving their presentations. Our findings that risk information was not presented suggest the bias may be limited. The requirement to get informed consent from pharmaceutical representatives may have limited participation in our study. We found that less than a quarter of Australian general practitioners who agreed to participate in the study returned the completed questionnaires. Among the 31 doctors who agreed to participate in the study, but did not return any completed questionnaires, more than half indicated that all pharmaceutical representatives declined to sign the consent form.

The validity of our results may be limited by the small sample size. The sample of general practitioners who participated in this study was not taken randomly from the population and the results might not be generalisable to all Australian and Malaysian doctors. Our findings were based on general practitioners' self reporting, which is also subject to bias in recall, however, the surveys were completed immediately after the encounter, so recall bias should have been limited. The generalisability of the findings was also limited by the low response rate. The findings may only be applicable to interactions between general practitioners and pharmaceutical representatives and could not be generalized to other specialties.

Healthy Skepticism subscribers involved in the study may be more critical of the quality of information provided by pharmaceutical representatives than other general practitioners. More sceptical general practitioners might be less easily convinced, asked pharmaceutical representatives' for more information; and recalled and reported the information in a different way. None of the general practitioners in Australia and 42% in Malaysia were from the same practices. Clustering within the practices could has affected the findings noted in Malaysia.

Given all Malaysian general practitioners who participated in the study were practicing in urban areas, our results could not be generalized to doctors in rural areas in Malaysia.

We only asked doctors to report the provision of approved product information sheets to them. In Australia, approved product information may be included in promotional materials given to doctors. Our findings on the provision of medicines information for type of main product discussed were limited by the sample.

## Conclusion

Information on indications and dosages were usually provided by pharmaceutical representatives in both Australia and Malaysia. However, risk and harmful effects of medicines were often missing in pharmaceutical representatives' presentations. This is a concern, as incomplete medicines information may lead to misrepresentation of the actual therapeutic value of the medicines and ignorance of safety issues may ultimately negatively impact on health outcomes.

## Competing interests

Two of the authors (NO and AV) are members of Healthy Skepticism, an international non-profit organisation aiming to improve health by reducing harm from misleading drug promotion. SBI and KO have been funded by several pharmaceutical companies to perform research, attend conferences and have received speaking honorariums.

## Authors' contributions

NO designed the study with input from AV and ER. NO applied for ethical approvals and recruited general practitioners in these two countries with the help of AV, ER, SBI and KO. NO gathered and interpreted the data and drafted the manuscript. NO, AV and ER were responsible for critical revision of the manuscript. All of the authors read and approved the final version submitted.

## Pre-publication history

The pre-publication history for this paper can be accessed here:

http://www.biomedcentral.com/1471-2458/10/743/prepub
